# Triple Elimination of HIV, Syphilis and HBV Mother‐To‐Child Transmission in Africa: From a WHO Initiative to a Respondent‐Level Reality

**DOI:** 10.1111/jvh.70274

**Published:** 2026-11-10

**Authors:** Erwan Vo‐Quang, Gibril Ndow, Lindsey Hiebert, Neil Gupta, Jana Manning, John Ward, Maud Lemoine

**Affiliations:** ^1^ MRC Unit The Gambia at London School of Hygiene & Tropical Medicine Banjul The Gambia; ^2^ Department of Gastroenterology and Digestive Oncology Georges Pompidou European Hospital Paris France; ^3^ Coalition for Global Hepatitis Elimination, Task Force for Global Health Decatur Georgia USA; ^4^ Division of Viral Hepatitis Centers for Disease Control and Prevention Atlanta Georgia USA; ^5^ Section of Hepatology, Division of Digestive Diseases, Department of Metabolism, Digestion and Reproduction Imperial College London London UK

In 2017, the World Health Organization (WHO) established the triple elimination initiative, aiming to end mother‐to‐child transmission (MTCT) of HIV, syphilis and hepatitis B virus (HBV) through integrated prevention, testing and treatment services within maternal and child health programmes [1].

Yet, the translation of this initiative into country‐level policy across the WHO African region remains poorly documented. To document the reported status of triple elimination policies and practices, we conducted a cross‐sectional rapid assessment among clinicians, policymakers and national programme officers working in the region, covering three domains: national strategic plans and guidelines, access barriers and out‐of‐pocket costs. The survey was disseminated electronically to 70 people through the Coalition for Global Hepatitis Elimination between 1 August 2024 and 1 March 2025 (Supplementary Material 1).

A total of 30 individuals (43% of those invited) completed the survey, reporting on 21 distinct countries of the WHO African region; seven countries were covered by more than one respondent. All proportions reported below are respondent‐level, and denominators refer to respondents rather than countries. Respondents were classified as doctors or nurses (50%), public health officers or health policymakers (33%), or other/unclassified (17%) and were based in East (40%), West (23%), Southern (17%), Central (13%) and North Africa (7%).

## 1 National Strategic Plans

46% of respondents (12/26) reported the existence of a national strategic plan addressing the triple elimination of MTCT of HIV, syphilis and HBV, while 54% (14/26) reported no existing plan (4 respondents were unable to confirm). Among those without an integrated plan, disease‐specific national strategic plans were more commonly reported: HIV‐specific plans by 11 respondents, HBV‐specific plans by 6, syphilis‐specific plans by 4 and a combined HIV/syphilis plan by 2. Despite this reported policy gap, 76% of respondents (19/25) reported that their Ministry of Health had prioritized integration of HBV services within HIV and syphilis programmes for the coming year, though fewer than half (43%, 10/23) confirmed that dedicated healthcare worker training to support this integration had been developed.

## 2 Clinical Practice Guidelines

Integrated national clinical practice guidelines on MTCT of HIV, syphilis and HBV were reported by 54% of respondents (14/26), with most having been developed or updated between 2020 and 2024. Among those without integrated guidelines, separate guidelines were reported for HIV (9 respondents), HBV (5) and syphilis (4). Regarding HBV‐specific antiviral prophylaxis, 41% (11/27) reported guidelines recommending antiviral prophylaxis for pregnant women with high HBV DNA levels or HBeAg positivity, 30% (8/27) a treat‐all approach for all HBsAg‐positive pregnant women regardless of viral load, and 15% (4/27) both strategies coexisting; a further 15% (4/27) reported no strategy in place. Timely hepatitis B birth dose vaccination, a cornerstone of the triple elimination strategy, was reported to be included in national guidelines by 73% of respondents (22/30), although its real‐life implementation was not assessed. When asked about planned adoption of the WHO 2024 conditional treat‐all recommendation, applicable in settings where HBV DNA and HBeAg testing are unavailable, 71% (20/28) indicated that adoption was planned in their setting, while 18% (5/28) reported having no existing HBV antiviral prophylaxis policy. Additionally, 73% of respondents (22/30) supported the management of HBV mono‐infected pregnant women within existing HIV clinic infrastructure.

## 3 Access Barriers

Significant barriers to HBV service delivery were reported. Among respondents able to answer, 70% (19/27) reported financial or logistical difficulties in accessing HBV rapid diagnostic tests, and 76% (22/29) reported similar barriers to Tenofovir‐based antiviral prophylaxis. Access to HBV viral load testing was not formally assessed in this survey; the high proportion of respondents anticipating adoption of the treat‐all strategy (71%) would be consistent with limited availability of HBV DNA testing, but this was not measured.

## 4 Out‐of‐Pocket Costs

Overall, 57% of respondents (17/30) reported that antenatal testing for all three infections was provided free of charge, while 37% (11/30) reported only partial coverage. HIV and syphilis treatment was reported to be fully subsidized for pregnant women by 80% of respondents (24/30), with partial coverage reported by the remaining 20% (6/30). In contrast, reported HBV treatment access was markedly more limited: 38% (10/26) reported free access to antiviral therapy (these reports came predominantly from respondents based in East Africa), 38% (10/26) reported out‐of‐pocket requirements, and 23% (6/26) indicated that no HBV antiviral therapy was available in their setting.

This rapid assessment, based on 30 respondents reporting on 21 countries, highlights important gaps in the implementation of the triple elimination strategy in the WHO African region. Fewer than half of respondents reported an integrated national strategic plan, and the clinical practice guidelines they described remain heterogeneous. These findings are consistent with, but not necessarily representative of, the broader regional picture. Access to HBV diagnostics and antiviral therapy was reported to face substantial financial and logistical barriers, in contrast to the relative availability of HIV and syphilis services, a contrast that may reflect decades of dedicated funding to fight HIV/AIDS in Africa. Among respondents, the anticipated adoption of the HBV treat‐all strategy is promising, but its feasibility and cost‐effectiveness in resource‐limited settings warrant rigorous evaluation. Several limitations should be acknowledged. This cross‐sectional rapid assessment relied on self‐reported data from a convenience sample recruited through a hepatitis elimination network, introducing a risk of sampling bias; respondents may not be representative of national programmes across the region and may overrepresent countries with more active hepatitis programmes. Data are respondent‐level reports and do not constitute a country‐level policy inventory: seven countries were represented by more than one respondent, and in three of them answers on the existence of an integrated national strategic plan diverged. The response rate was 43% (30/70); non‐responders were not characterized, so the direction of any resulting bias cannot be determined, and participation was uneven across subregions, with East Africa contributing the largest share of respondents. Survey responses were not triangulated with external sources such as published national guidelines or WHO policy tracking databases. Accordingly, findings should be interpreted as indicative rather than definitive, and may not be generalizable to the broader WHO African region. Validated, nationally representative assessments are needed to confirm these observations. Nevertheless, this survey highlights that major additional efforts are needed to reach the triple elimination goal in Africa.

## Conflicts of Interest

The authors declare no conflicts of interest.

## Supporting information


**Supplementary Material 1.** Questionnaire used in the cross‐sectional rapid assessment of triple elimination policies and practices in the WHO African region.

## Data Availability

The data that support the findings of this study are available on request from the corresponding author. The data are not publicly available due to privacy or ethical restrictions.

## References

[1] World Health Organization , Triple Elimination Initiative of Mother‐To‐Child Transmission of HIV, Syphilis and Hepatitis B (WHO, 2017), https://www.who.int/initiatives/triple‐elimination‐initiative‐of‐mother‐to‐child‐transmission‐of‐hiv‐syphilis‐and‐hepatitis‐b.

