# Supplementary material for: Triple Elimination of HIV, Syphilis and HBV Mother‐To‐Child Transmission in Africa: From a WHO Initiative to a Respondent‐Level Reality
Source: J Viral Hepat. 2026 Nov 10;33(12):e70274. doi: 10.1111/jvh.70274 (PMC13752450; doi:10.1111/jvh.70274)
Supplement: Supplementary file 1 [file JVH-33-0-s001.docx]

**Supplementary Material 1.** Questionnaire used in the cross-sectional rapid assessment of triple elimination policies and practices in the WHO African region

Note: The questionnaire was disseminated electronically to 70 clinicians, policymakers and national programme officers through the Coalition for Global Hepatitis Elimination between 1 August 2024 and 1 March 2025. It collected respondent characteristics (country and occupation) and covered three domains: Section A, national strategic plans and clinical practice guidelines on mother-to-child transmission of HIV, syphilis and HBV (Q1–Q7); Section B, testing and management of HIV, syphilis and HBV in pregnant women, including barriers to access to tenofovir (Q8–Q12); and Section C, financing of care, i.e. out-of-pocket costs for testing and treatment (Q13–Q14).

*Triple elimination initiative*

**Survey**

*The WHO recommends integrating HBV services alongside HIV and syphilis services in antenatal care. This approach, known as the "Triple Elimination Initiative," aims to reduce mother-to-child transmission of HBV in resource-limited countries. The following questions will help us describe the policy related to this strategy in sub-Saharan Africa. We would be grateful if you could provide answers to the following questions.*

- Name of the participant: ……………………………………………………………..……………………………………..
- Age: …………………………………………………………………………………………………………………………………….
- Sex: ……………………………………………………………………………………………………………………………………..
- Location/country: ……………………………………………………………………………………………………………….
- Occupation: …………………………………………………………………………………………………………………………

# 1. SECTION A: National strategic plans and clinical practice guidelines

- Q1. Is there a national strategic plan in your country that addresses mother-to-child transmission of HIV, syphilis, and hepatitis B?

Yes	No	I do not know

- Q1.1. If yes, when was the national strategic plan implemented? (years)

…

- Q1.1. If no, are there separate national strategic plans available for each of the following infections: HIV, syphilis, and Hepatitis B?

Yes	No	I do not know

- Q1.1.1. If yes, when was each national strategic plan implemented? (years)

HIV national strategic plan: …

Syphilis (or STI) national strategic plan: …

Hepatitis B national strategic plan: …

- Q2. Is there a clinical practice guideline on the topic of mother-to-child transmission of HIV, syphilis, and hepatitis B available in your country?

Yes	No	I do not know

- Q2.1. If yes, when was the clinical practice guideline on the topic of mother-to-child transmission of HIV, syphilis, and hepatitis B implemented? (years)

…

- Q2.2. If no, are there separate clinical practice guidelines available for each of the following infections: HIV, syphilis, and Hepatitis B?

Yes	No	I do not know

- Q2.2.2 If yes, when was each clinical practice guidelines implemented? (years)

HIV clinical practice guidelines: …

Syphilis (or STI) clinical practice guidelines: …

Hepatitis B clinical practice guidelines: …

- Q3. Is simultaneously screening for HIV, syphilis, and HBV recommended during antenatal care at all health facility levels, regardless of whether they are located in rural or urban areas?

Yes	No	I do not know

- Q4. Is the Birth Dose Hepatitis B vaccine strategy included in the national strategic plan??

Yes	No	I do not know

- Q5. Is the Birth Dose Hepatitis B vaccine strategy included in the clinical practice guidelines?

Yes	No	I do not know

- Q6. Is Tenofovir (HBV antiviral therapy) recommended for pregnant mothers with high viral loads?

Yes	No	I do not know

- Q7. Does COVID-19 pandemic impact on decisions to introduce or scale-up HBV integration with HIV and syphilis services?

Yes	No	I do not know

# 2. SECTION B: HIV, syphilis and HBV testing and management in pregnant mothers

- Q8. Is testing for any of the three infections conducted using Point-of-Care (POC) tests or at the laboratory in most health facilities?

Point-of-Care (POC) tests	laboratory

- Q9. If Tenofovir is recommended for pregnant mothers with high viral loads, do you experience any financial difficulties in accessing Tenofovir?

Yes	No	I do not know	Not applicable

- Q10. If Tenofovir is recommended for pregnant mothers with high viral loads, do you encounter any logistical difficulties in accessing Tenofovir?

Yes	No	I do not know	Not applicable

- Q11. In urban health facilities, is care for pregnant women infected with HBV provided within the same facility?

Yes	No	I do not know

- Q12. In rural health facility, is care for pregnant women infected with HBV provided within the same facility?

Yes	No	I do not know

# 3. Section C: Financing in care

- Q13. Is testing for any of the three targeted infections (HIV, syphilis, and HBV) free for pregnant mothers attending health facilities?

Yes	No	I do not know

- If testing for any of the three targeted infections (HIV, syphilis, and HBV) is not free, what test incurs an out-of-pocket cost?

…

- Q14. Is treatment for any of the three targeted infections (HIV, syphilis, and HBV) provided free of charge for pregnant mothers who attend health facilities?

Yes	No	I do not know

- If treatment for any of the three targeted infections (HIV, syphilis, and HBV) is not free, what test incurs an out-of-pocket cost?

…

*Thank you for responding to the previous questions. We kindly request access to the national strategic plan and guidelines. If possible, please share these documents with us via email. Thank you in advance for your cooperation.*
